# The Role of Local and General Anaesthesia in Modern Dental Practice: Clinical Decision-Making Frameworks, Patient Selection, Trends, Challenges, and Outcomes

**DOI:** 10.7759/cureus.105824

**Published:** 2026-03-25

**Authors:** Vaibhav Anand, Solanki Sohini Nareshkumar, Ena Maakhni, Kumari Priyanka Shrivastava, Vikram Arora, Jyoti Mathur

**Affiliations:** 1 Department of Oral and Maxillofacial Surgery, Uttar Pradesh University of Medical Sciences, Saifai, IND; 2 Department of Anaesthesiology, Dr. Panjabrao Deshmukh Memorial Medical College, Amravati, IND; 3 Department of Dentistry, All India Institute of Medical Sciences, Gorakhpur, Gorakhpur, IND; 4 Department of Oral Pathology and Microbiology, Rajasthan University of Health Sciences (RUHS), Jaipur, IND; 5 Department of Public Health Dentistry, ITS Dental College, Hospital and Research Centre, Greater Noida, IND; 6 Department of Pedodontics and Preventive Dentistry, Dharmsinh Desai University, Nadiad, IND

**Keywords:** anaesthesia techniques, dental practice, monitoring standards, patient outcomes, pharmacological innovations

## Abstract

Anaesthesia is essential in modern dental practice for ensuring patient comfort and enabling safe and effective dental procedures. Increasing procedural complexity, a growing population of medically vulnerable patients, and higher expectations for safe outpatient care intensify the demand for anaesthetic approaches aligned with contemporary clinical standards. Despite substantial advancements in pharmacology and monitoring, inconsistencies persist in clinical decision-making, patient selection criteria, and the integration of safety standards across diverse dental settings, creating variability in practice and outcomes. This review aims to synthesise updated knowledge on local and general anaesthesia in dentistry, with emphasis on techniques, pharmacological principles, clinical challenges, and emerging innovations influencing patient outcomes. A targeted search of major scientific databases is conducted to identify literature published between 2015 and 2025, using predefined inclusion and exclusion criteria to ensure relevance and currency. The narrative review integrates evidence related to patient selection, perioperative monitoring requirements, complication profiles, and considerations for high-risk populations. Technological developments, including ultrasound-guided techniques, computer-assisted delivery systems, and sustained-release local anaesthetic formulations, are examined for their role in improving precision and safety. Ethical considerations, informed consent, and global practice standards are addressed to provide a comprehensive perspective on current practice. Advances in anaesthetic pharmacology, monitoring, and delivery strengthen procedural safety and patient experience in dental care. Continued research, along with wider adoption of standardised guidelines, remains necessary to improve consistency, safety, and quality of dental anaesthetic practice across diverse clinical settings worldwide.

## Introduction and background

Variability in clinical decision-making, patient risk stratification, and safety standards across dental settings continues to influence anaesthetic choice and perioperative outcomes, underscoring the need for clearer integration of contemporary evidence into routine practice [1]. Anaesthesia plays a central role in modern dental care, enabling complex procedures through modulation of pain perception, patient awareness, and physiological responses to procedural stress [1]. Advances in anaesthetic pharmacology and delivery systems have improved the safety and reliability of both local and general anaesthesia, allowing more individualised approaches tailored to patient and procedural factors [2]. Local anaesthesia involves reversible loss of sensation in a defined anatomical region without loss of consciousness, whereas general anaesthesia produces a controlled state of unconsciousness that requires advanced airway management and physiological monitoring. Local anaesthesia remains the primary modality in routine dental practice due to its rapid onset, site-specific action, and predictable recovery profile [3]. In contrast, general anaesthesia is reserved for extensive oral surgical procedures, patients with severe behavioural or cognitive limitations, and situations requiring complete unconsciousness to ensure procedural precision and patient safety [4]. Technological developments, including advanced airway devices, depth-of-anaesthesia monitoring systems, and multimodal analgesic strategies, are increasingly integrated into clinical practice and contribute to improved perioperative safety [5].

Despite these developments, important challenges continue to affect the safety and predictability of dental anaesthesia. Interindividual anatomical, metabolic, and genetic variability creates uncertainty in dosing requirements, onset times, and toxicity risk, particularly in patients with chronic systemic disease, polypharmacy, or reduced physiological reserve [2,6]. The increasing proportion of elderly and medically compromised individuals further emphasises the need for structured preoperative assessment frameworks capable of capturing multifactorial risk profiles [7]. Inequitable access to advanced monitoring technologies - such as capnography, continuous oxygenation assessment, cardiovascular monitoring, and computer-assisted delivery systems - limits early recognition of complications, including local anaesthetic systemic toxicity (LAST), airway obstruction, and haemodynamic instability [4]. Differences in simulation-based training, emergency preparedness, and adherence to international anaesthesia safety standards also contribute to variability in care quality [8]. Moreover, anaesthetic selection pathways remain insufficiently standardised, and many guidelines lack procedure-specific criteria defining thresholds for escalation from local to general anaesthesia, monitoring requirements, and risk stratification parameters at patient, procedural, and institutional levels [6]. Although patient anatomy, procedure duration, anxiety levels, comorbidities, and airway characteristics inform anaesthetic choice, clinician-dependent variation persists in how these factors are weighted across levels of clinical complexity [9]. Taken together, these gaps highlight the need for clearer, evidence-informed decision frameworks to promote consistency and safety across care settings [3].

Special patient populations introduce further complexity. Individuals with cardiovascular disease, pulmonary disorders, endocrine dysfunction, coagulation abnormalities, neurocognitive impairment, or pregnancy exhibit altered pharmacokinetic and pharmacodynamic responses that complicate anaesthetic planning [10]. Paediatric patients present additional challenges due to distinct airway anatomy, developmental pharmacology, and increased sensitivity to sedative and anaesthetic agents [2]. Current evidence addressing these groups is often dispersed across pharmacological, procedural, and outcome-focused studies, limiting the development of integrated clinical strategies [11]. At the same time, technological and pharmacological innovations continue to expand therapeutic possibilities, although their consistent implementation in routine dental practice remains uneven [5]. Buffered local anaesthetic formulations, needle-free delivery systems, sustained-release anaesthetics, and computer-controlled dosing platforms may enhance comfort and reduce adverse events, yet large-scale clinical validation is still limited [12]. Advances in general anaesthesia safety infrastructure - including sophisticated airway devices, automated infusion systems, and depth-of-anaesthesia monitoring with alarm integration - improve physiological control and risk mitigation; however, their uptake within dental settings varies according to institutional capacity and clinical context [3]. Further evaluation is required to determine their impact on perioperative stability, recovery trajectories, and functional outcomes in dental populations [8].

Ethical and medico-legal considerations represent an essential component of contemporary anaesthetic practice. Expectations regarding transparency, documentation, and informed consent increasingly influence procedural planning in dental anaesthesia [13]. Emphasis on patient safety has extended standards for anaesthetic competency, credentialing, and facility preparedness within outpatient dental environments. Persistent disparities in access to safe anaesthesia services across regions and populations reinforce the obligation to ensure equitable care for vulnerable groups. Collectively, these clinical, technological, and ethical considerations support the development of a coherent evaluative framework integrating pharmacological principles, technical considerations, risk stratification, monitoring standards, patient-centred outcomes, and emerging innovations. This review therefore offers a structured and comparative analysis of local and general anaesthesia in dental practice, focusing on indications, safety considerations, patient selection parameters, and evolving developments that shape contemporary clinical outcomes. A comprehensive appraisal of these elements is essential to optimise anaesthetic decision-making and enhance the safety and effectiveness of dental care across diverse clinical contexts.

Objectives of the review

This review presents an integrated assessment of local and general anaesthesia in dental practice, with emphasis on current techniques, unresolved clinical challenges, and implications for patient outcomes. Specifically, it aims to (1) compare clinical indications and patient selection criteria for local versus general anaesthesia; (2) evaluate safety protocols, monitoring standards, and risk stratification approaches across different care settings; and (3) examine emerging pharmacological and technological innovations that influence procedural precision and patient-centred outcomes. Through this structured analysis, the review seeks to clarify areas of variability in practice and support more consistent, evidence-informed anaesthetic decision-making. It further synthesises existing evidence to promote safe and high-quality anaesthetic care across diverse clinical contexts.

Methodology

Study Design

This study was conducted as a narrative review to provide a structured qualitative synthesis of contemporary evidence on local and general anaesthesia in dental practice. The objective was to integrate findings from heterogeneous study designs without quantitative pooling, meta-analysis, or comparative statistical modelling.

Data Sources and Search Strategy

A structured literature search was performed in PubMed, Scopus, Web of Science, and Google Scholar to identify relevant publications. The search timeframe was limited to studies published between January 2015 and December 2025 to ensure contemporary relevance. The search strategy incorporated combinations of keywords and Boolean operators related to local anaesthesia, general anaesthesia, dental or oral surgery anaesthesia, anaesthetic techniques, pharmacology, pharmacokinetics, monitoring standards, safety, complications, and patient outcomes. Searches were restricted to peer-reviewed publications published in English. In addition to electronic database searches, manual screening of reference lists of eligible studies was undertaken to identify additional relevant articles.

Eligibility Criteria

Inclusion criteria: Studies were eligible for inclusion if they consisted of clinical research, such as randomised controlled trials, cohort or observational studies, systematic reviews or meta-analyses, and evidence-based guidelines or consensus statements specifically addressing anaesthetic techniques, pharmacology, safety, monitoring, or outcomes in dental settings.

Exclusion criteria: Studies were excluded if they involved non-dental procedures, focused on obsolete anaesthetic agents no longer in clinical use, were conducted exclusively in non-human models, lacked sufficient methodological detail, or were published outside the predefined timeframe of 2015-2025.

Study Selection and Data Handling

Titles and abstracts were screened for relevance to dental anaesthesia practice. Full-text articles of potentially eligible studies were subsequently reviewed to confirm suitability for inclusion. Data were organised and synthesised thematically across major domains, including pharmacology, clinical techniques, safety protocols, patient selection, complications, high-risk populations, and emerging technologies.

Risk of Bias and Quality Assessment

Although the review was conducted using a narrative design, the methodological quality of included studies was evaluated through a structured appraisal process. Two independent authors assessed eligible studies for clarity of methodology, appropriateness of study design, sample characteristics, outcome reporting transparency, and consistency of findings. Any disagreements were resolved through discussion to reach a consensus.

Data Synthesis

Findings were synthesised descriptively and analytically to identify patterns, areas of agreement, sources of variability, and gaps in current knowledge. No meta-analysis, heterogeneity testing, or publication bias assessment was undertaken, as the purpose of the review was conceptual integration rather than statistical aggregation.

## Review

Pharmacological basis of dental anaesthesia

The pharmacological and pharmacokinetic properties of anaesthetic agents used in dental practice are determined by molecular mechanisms, distribution characteristics, and physiological interactions that shape their clinical efficacy and safety profiles [14]. Local anaesthetic agents act by reversibly blocking voltage-gated sodium channels in peripheral nerves, thereby preventing the initiation and propagation of action potentials. This interruption of nociceptive transmission to the central nervous system permits targeted pain control with limited systemic involvement [13]. Variations in lipid solubility, protein binding, and pKa, among agents, such as lidocaine, articaine, mepivacaine, and bupivacaine, account for differences in potency, onset time, and tissue penetration [8]. Vasoconstrictors are commonly co-administered to prolong anaesthetic duration and reduce systemic absorption, thereby improving procedural control; however, cautious use is required in patients with cardiovascular compromise [2].

**Figure 1:**
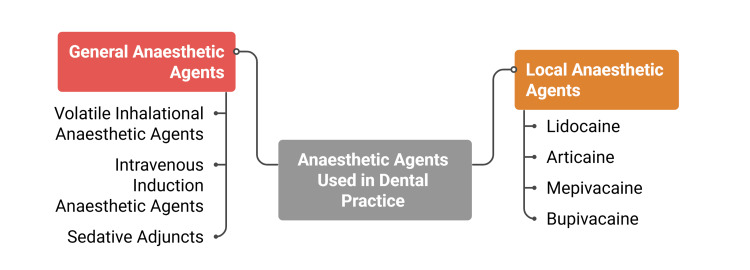
Anaesthetic Agents Used in Dental Practice Image created by the authors using Napkin AI (Los Altos, CA)

General anaesthetic agents used in dental settings primarily modulate central nervous system activity by enhancing inhibitory neurotransmission or suppressing excitatory signalling pathways [15]. Volatile agents, intravenous induction drugs, and sedative adjuncts exert their effects through interaction with gamma-aminobutyric acid (GABA) receptors, N-methyl-D-aspartate (NMDA) receptors, and related molecular targets [6]. The degree of central nervous system depression varies according to patient-specific factors, including age, comorbidities, pharmacokinetic variability, and concurrent medication use, resulting in differences in anaesthetic requirements across populations [9]. Toxicity thresholds represent a critical consideration for both local and general anaesthetic techniques. Elevated plasma concentrations of local anaesthetic agents may produce neurological symptoms, such as tinnitus, circumoral anaesthesia, seizures, and, in severe cases, cardiovascular collapse due to myocardial depression and conduction disturbances [4]. General anaesthetic agents carry additional risks, including airway compromise, haemodynamic instability, and postoperative cognitive changes. Because cardiovascular and neurological responses largely determine perioperative risk, precise dosing, continuous physiological monitoring, and individualised agent selection are essential for maintaining safety and procedural stability in dental practice [16]. The major types of anaesthetic agents used in dentistry are summarised in Figure 1.

Contemporary local anaesthesia techniques in dental practice

Contemporary local anaesthesia techniques in dental practice encompass a range of approaches tailored to specific clinical scenarios, with emphasis on procedural precision, patient comfort, and reliability [17]. Conventional infiltration involves deposition of anaesthetic solution near terminal nerve branches and is routinely used to achieve pulpal and soft tissue anaesthesia in the maxilla and in selected mandibular regions where bone porosity permits adequate diffusion [8]. Nerve block techniques expand the field of anaesthesia by targeting major neural trunks, thereby providing broader and deeper regional analgesia. Common blocks - including buccal, mental, long buccal, inferior alveolar, and anterior superior alveolar nerve blocks - are widely applied in restorative, surgical, and endodontic procedures due to their extensive coverage [12].

When conventional approaches fail to achieve sufficient pulpal anaesthesia or when reduction of soft tissue involvement is desirable, supplementary techniques may be indicated. Periodontal ligament injections provide highly localised pulpal anaesthesia and are particularly useful when standard nerve blocks are contraindicated. Delivery under controlled pressure into the periodontal ligament space allows rapid onset with minimal drug volume, improving efficiency and patient tolerance [18]. Intraosseous and intraseptal injections further enhance anaesthetic efficacy by depositing the solution directly into cancellous bone, facilitating reliable pulpal anaesthesia in dense mandibular regions [4]. Advances in image-guided and computer-assisted delivery systems improve consistency in needle positioning, flow rate control, and operator feedback [9]. These refinements enhance solution dispersion, reduce discomfort, and improve predictability across varying anatomical conditions [19]. An overview of principal targets, indications, and advantages of commonly used local anaesthesia techniques is presented in Table 1.

**Table 1:** Local Anaesthesia Techniques in Dentistry IAN: Inferior Alveolar Nerve, PSA: Posterior Superior Alveolar, PDL: Periodontal Ligament, IO: Intraosseous, CAD: Computer-Assisted Delivery

Technique	Primary Target Site	Typical Clinical Use	Advantages	References
Infiltration	Terminal nerve branches	Routine restorative and minor surgical procedures	Rapid onset, minimal complexity	[18]
Nerve Block	Major neural trunks (e.g., IAN, PSA)	Extensive restorative, endodontic, and surgical procedures	Broad regional coverage	[20]
PDL Injection	Periodontal space	Localised pulpal anaesthesia	Limited soft-tissue effect, rapid action	[7]
IO Injection	Cancellous bone	Anaesthesia in dense mandibular regions	Fast onset, effective in resistant cases	[11]
CAD	Site-specific targeted delivery	Precision-focused procedures	Controlled flow, enhanced comfort	[14]

General anaesthesia approaches for dental procedures

General anaesthesia in dental practice provides a controlled state of unconsciousness, effective analgesia, and complete immobility, making it particularly suitable for procedures requiring extensive surgical access, secure airway control, and optimal operative conditions for precision [21]. Induction is most commonly achieved intravenously, allowing rapid and predictable transition to an anaesthetised state with muscle relaxation and attenuation of autonomic responses [13]. Anaesthesia is maintained using target-controlled infusion systems or volatile agents, enabling continuous titration to sustain an appropriate depth according to patient physiology and procedural demands [22].

Airway management remains a central safety consideration during dental procedures under general anaesthesia. The proximity of the operative field to the airway increases the risk of obstruction and aspiration, necessitating careful preoperative planning and protective strategies [18]. Selection among endotracheal intubation, laryngeal mask airway, or nasotracheal intubation depends on anatomical characteristics, surgical access requirements, and individual patient risk profiles. Supportive measures - including optimal positioning, continuous suction, and airway humidification - contribute to maintaining effective ventilation and gas exchange throughout the procedure [16].

Monitoring of anaesthetic depth facilitates precise control of central nervous system depression and reduces the likelihood of intraoperative instability [5]. Electroencephalographic indices, processed neural monitoring, and haemodynamic parameters provide objective data that guide timely adjustment of drug administration to preserve physiological balance [21]. Continuous surveillance of oxygenation, ventilation, and cardiovascular status remains essential, particularly in patients with systemic comorbidities [17]. In dental settings, general anaesthesia is typically indicated for prolonged maxillofacial surgery, severe dental anxiety or phobia, limited patient cooperation, complex medical conditions, and heightened airway sensitivity [22]. The selection of anaesthetic technique is guided by therapeutic objectives, anticipated procedural duration, and expected physiological stress. Post-anaesthetic recovery requires structured assessment of airway patency, neurological status, pain control, and haemodynamic stability to ensure safe discharge to ambulatory or inpatient care [14]. Integration of induction, maintenance, vigilant monitoring, and structured recovery protocols ensures that general anaesthesia supports both procedural quality and patient safety during complex dental interventions [20].

Patient selection criteria for local versus general anaesthesia

The choice between local and general anaesthesia in dental practice is based on a structured evaluation of physiological status, behavioural profile, procedural complexity, and anticipated response to anaesthetic exposure [20]. Clinical decision-making begins with a comprehensive assessment of systemic health, including cardiovascular, respiratory, endocrine, neurological, and hepatic functions, as these factors directly influence drug metabolism, haemodynamic stability, and airway safety [17]. The American Society of Anesthesiologists (ASA) physical status classification is widely applied to stratify perioperative risk and guide anaesthetic planning [23]. Patients within lower ASA categories are generally suitable for local anaesthesia, whereas those with higher classifications may require general anaesthesia with enhanced monitoring and airway protection due to elevated perioperative risk [8].

Behavioural considerations also significantly influence anaesthetic selection. Severe dental anxiety, phobic responses, communication barriers, and limited cooperation can compromise the effectiveness of local techniques and increase the likelihood of movement, restricted access, and incomplete treatment [15]. In such cases, general anaesthesia provides a controlled and predictable environment, particularly for patients with sensory sensitivities, cognitive impairment, or neurodevelopmental disorders [21]. Age-related factors further shape planning. Paediatric patients may present challenges related to anatomical development, limited procedural understanding, and heightened emotional responses, which can necessitate general anaesthesia for extensive or invasive interventions [12]. Conversely, geriatric patients frequently exhibit polypharmacy, frailty, altered pharmacokinetics, and reduced physiological reserve, requiring careful titration of local agents or, when indicated, general anaesthesia with intensified monitoring.

**Figure 2:**
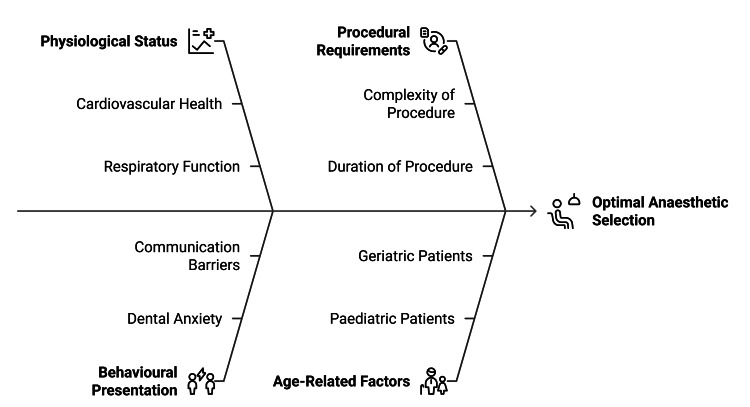
Clinical Factors Influencing Anaesthetic Selection in Dental Practice Image created by the authors using Napkin AI (Los Altos, CA)

Procedural complexity remains a critical determinant of anaesthetic choice [24]. Local anaesthesia is typically appropriate for minor restorative procedures and limited surgical interventions, whereas prolonged maxillofacial surgery, airway-involved procedures, or operations of significant duration may justify general anaesthesia. By systematically integrating systemic health, behavioural factors, age-related considerations, and procedural demands, clinicians can individualise anaesthetic strategies to optimise safety, efficiency, and treatment outcomes in dental practice [25]. Figure 2 illustrates representative clinical scenarios that assist in determining the appropriate modality based on anxiety level, procedural complexity, duration, and associated risk.

Safety protocols and monitoring standards in dental anaesthesia

Safety protocols, including monitoring standards and hazard preparedness measures, form the foundation of anaesthetic practice in dentistry and support early detection of physiological instability, thereby reducing adverse events [26]. A comprehensive pre-anaesthetic assessment establishes the patient's physiological baseline and overall perioperative risk profile through systematic evaluation of cardiovascular and respiratory status, medication use, allergy history, and potential airway difficulties. This assessment guides individualised dose planning and monitoring intensity, particularly in patients with limited physiological reserve or complex comorbidities [24]. Intraoperative safety relies on continuous monitoring of oxygenation, ventilation, circulation, and anaesthetic depth. Pulse oximetry provides real-time measurement of arterial oxygen saturation and serves as an early indicator of hypoxaemia [18]. Capnography supports evaluation of ventilatory adequacy and confirmation of airway patency, while blood pressure monitoring and electrocardiography enable ongoing assessment of haemodynamic stability and early identification of arrhythmias or circulatory changes [9].

Monitoring anaesthetic depth allows precise titration of agents during general anaesthesia and reduces the risks of both inadequate sedation and excessive cardiorespiratory depression. Immediate availability of emergency equipment - including suction devices, airway adjuncts, emergency medications, and defibrillators - is essential for rapid management of unexpected complications [21]. Post-anaesthetic recovery monitoring represents a structured continuation of intraoperative surveillance and includes repeated assessment of consciousness level, airway patency, respiratory adequacy, haemodynamic stability, and pain control until predefined recovery criteria are met [27]. Standardised discharge and transition protocols ensure that patients fulfil established safety benchmarks prior to transfer or discharge, including stable vital signs, adequate oxygenation, and satisfactory recovery from immediate post-anaesthetic effects. Consistent application of these measures minimises perioperative risk across diverse dental care settings [28]. Table 2 summarises key physiological parameters, their clinical relevance, and the monitoring modalities required for safe delivery of dental anaesthesia.

**Table 2:** Core Monitoring Components in Dental Anaesthesia SpO₂: Peripheral Oxygen Saturation, EtCO₂: End-Tidal Carbon Dioxide, BP: Blood Pressure, ECG: Electrocardiogram, DOA: Depth of Anaesthesia, EEG: Electroencephalogram

Monitoring Parameter	Primary Function	Clinical Significance	Monitoring Method	References
SpO₂	Oxygenation assessment	Early detection of hypoxia	Pulse oximetry	[17]
EtCO₂	Ventilation assessment	Confirmation of airway patency	Capnography	[28]
BP	Circulatory evaluation	Identification of haemodynamic fluctuation	Non-invasive BP monitor	[21]
ECG	Cardiac rhythm assessment	Recognition of arrhythmias	ECG leads	[12]
DOA	Depth assessment	Prevention of anaesthetic under-/overdosage	Neural or EEG-based indices	[25]

Complications associated with local and general anaesthesia in dentistry

Complications associated with dental anaesthesia result from interactions between pharmacological effects, anatomical variation, and individual physiological responses and may compromise patient safety before, during, or after dental procedures [29]. Although local anaesthetic techniques are generally safe, adverse events, such as allergic reactions, soft tissue injury, haematoma formation, and transient neurological disturbances, can occur [5]. LAST is a rare but potentially life-threatening complication caused by elevated plasma concentrations that depress central nervous system activity and impair myocardial conduction [30]. Early symptoms include tinnitus, dizziness, and circumoral numbness, which may progress to seizures or cardiovascular collapse in severe cases. Injections administered near major neural structures may lead to neurapraxia or altered sensation, with recovery depending on the extent of neural injury [22].

General anaesthesia introduces additional risks related to airway management, cardiovascular regulation, and depth of central nervous system depression [14]. Airway obstruction is more likely in patients with reduced anatomical space, obesity, or craniofacial abnormalities, creating challenges for ventilation and gas exchange. Intraoperative complications may include laryngospasm, aspiration, and displacement of airway devices, particularly during procedures that restrict oral access [27]. Systemic vasodilation can contribute to haemodynamic instability or myocardial depression, especially in individuals with limited cardiovascular reserve. Continuous monitoring of blood pressure, heart rate, and oxygen saturation facilitates early detection of instability and prompt intervention [16]. Post-anaesthetic complications may involve transient cognitive changes, delayed recovery, nausea, vomiting, and impaired coordination, particularly in elderly patients or those with systemic disease [19]. Local anaesthetic administration may also result in soft tissue trauma, trismus, or prolonged numbness, especially in regions with dense connective tissue or complex innervation [24]. Effective complication prevention depends on thorough preoperative assessment, awareness of patient-specific risk factors, procedural complexity, and potential drug interactions, combined with vigilant intraoperative and postoperative monitoring [16].

Pain management and post-anaesthetic recovery outcomes

Effective pain control and smooth recovery are central to successful dental treatment outcomes. Adequate management of nociceptive input promotes early functional recovery, reduces postoperative discomfort, and improves tolerance of daily activities [31]. Local anaesthetic agents provide immediate postoperative analgesia through sustained peripheral nerve blockade, with duration influenced by agent selection, tissue vascularity, and adjunctive vasoconstrictor use [5]. As anaesthetic effects subside, non-steroidal anti-inflammatory drugs, acetaminophen, and multimodal analgesic approaches support control of inflammatory pain, preserve oral function, and reduce reliance on opioid therapy [29].

Recovery outcomes depend on anaesthetic modality, physiological reserve, procedural duration, and individual pharmacokinetic variability. General anaesthesia may be associated with transient cognitive slowing, impaired coordination, and delayed psychomotor recovery [30]. Structured postoperative observation is therefore required to ensure safe transition to ambulatory care. Restoration of airway reflexes, haemodynamic stability, and absence of residual sedation indicate readiness for discharge. Supportive interventions, including supplemental oxygen, intravenous fluids, and gradual mobilisation, assist physiological stabilisation during emergence [24].

Postoperative sensory and motor function of oral tissues provides additional indicators of recovery quality. Persistent numbness may impair mastication, increase the risk of soft tissue injury, and diminish protective reflexes [20]. Temporary trismus or muscular discomfort can occur after prolonged procedures or sustained mouth opening. Patient-reported outcomes offer valuable insight into comfort, perceived recovery quality, and adherence to postoperative guidance. Higher satisfaction is associated with effective analgesia, minimal nausea, and earlier return to normal function [18]. Optimising recovery requires coordinated intraoperative management, targeted analgesic strategies, and individualised postoperative assessment to support stable recovery trajectories and durable treatment outcomes [32].

Special considerations for high-risk populations in dental anaesthesia

High-risk patient populations present distinct physiological and anatomical considerations that influence anaesthetic planning, dose selection, and monitoring requirements in dental practice. Patients with unstable or complex medical conditions often exhibit altered pharmacokinetics, reduced organ reserve, and increased susceptibility to adverse reactions, necessitating individualised anaesthetic strategies [33]. Cardiovascular disease increases the likelihood of haemodynamic fluctuations during both local and general anaesthesia, requiring careful selection of vasoconstrictor concentration and vigilant cardiovascular monitoring [31]. Respiratory disorders further complicate management due to compromised airway patency, reduced ventilatory capacity, and heightened sensitivity to sedative agents, making maintenance of effective gas exchange essential throughout treatment [22].

Endocrine conditions, such as diabetes and thyroid disorders, modify metabolic responses to anaesthetic agents and may impair tissue healing [7]. Perioperative planning must therefore address stress responses, glycaemic variability, and potential drug interactions through structured evaluation and optimisation. Hepatic or renal impairment can delay elimination of anaesthetic agents, increasing the risk of accumulation and toxicity [26]. In these patients, dose adjustment and extended postoperative observation are necessary to reduce complications.

Patients with neurodevelopmental or cognitive disorders often demonstrate limited procedural tolerance, behavioural unpredictability, sensory hypersensitivity, and communication challenges [17]. General anaesthesia or advanced sedation may be required to ensure procedural safety and patient comfort. Supportive measures - including careful positioning, behavioural guidance, and reduction of environmental stimuli - further enhance safety [25]. Pregnancy also requires tailored anaesthetic planning, as physiological changes such as increased blood volume, altered respiratory mechanics, and heightened susceptibility to hypoxia influence agent selection [27]. Agents with favourable safety profiles and minimal placental transfer are preferred, along with positioning strategies that support maternal and foetal oxygenation [31]. A comprehensive medical history, evaluation of potential drug interactions, and review of previous anaesthetic responses remain essential components of safe management in high-risk dental patients [24].

Technological and pharmacological innovations in dental anaesthesia

Advances in technology and pharmacology continue to refine the precision, comfort, and safety of anaesthetic delivery in dental practice [34]. Improvements in formulation science have produced local anaesthetic agents with enhanced pharmacokinetic profiles, supporting longer duration of action, reduced systemic absorption, and fewer adverse effects. Sustained-release formulations allow controlled diffusion within targeted tissues, thereby extending postoperative analgesia without repeated dosing [21]. Buffered anaesthetic solutions improve tissue compatibility by adjusting pH closer to physiological levels, leading to faster onset and reduced injection discomfort with more consistent clinical performance [15].

Computer-assisted delivery systems have further improved local anaesthetic administration. By controlling injection pressure and flow rate, these systems reduce tissue trauma and promote more uniform anaesthetic dispersion [32]. This controlled delivery increases the predictability of nerve blockade and lowers peak plasma concentrations, thereby decreasing the risk of systemic toxicity. Such systems are particularly advantageous in paediatric and anxious patients, where gradual administration minimises discomfort and involuntary movement [34]. Imaging and monitoring innovations also enhance procedural safety. Ultrasound-guided techniques permit real-time visualisation of anatomical structures, improving needle placement accuracy and reducing the likelihood of neural or vascular injury [30]. Advanced monitoring technologies - including enhanced capnography, near-infrared spectroscopy, and refined depth-of-anaesthesia indices - provide detailed physiological data during general anaesthesia, enabling early identification of instability and tailored anaesthetic titration in medically complex patients [35].

Recent pharmacological developments prioritise agents with rapid onset, lower cardiotoxic potential, and improved metabolic clearance. Structural modifications and reduced lipid solubility decrease systemic retention and central nervous system penetration [16]. Adjunctive receptor-targeted compounds enhance anaesthetic effectiveness by modulating peripheral sensitisation and synergistic neural pathways [28]. Together, these advances reflect a continued movement toward more precise, individualised, and safety-oriented anaesthetic strategies in contemporary dental practice. Table 3 summarises key developments in anaesthetic formulations, delivery systems, and imaging technologies that support safer and more accurate dental care.

**Table 3:** Key Innovations in Contemporary Dental Anaesthesia CAD: Computer-Assisted Delivery, USG: Ultrasound Guidance

Innovation Type	Representative Examples	Primary Benefit	Clinical Application	References
Sustained-Release Formulations	Long-acting local anaesthetics	Prolonged analgesia	Postoperative pain management	[27]
Buffered Solutions	pH-adjusted anaesthetics	Faster onset, reduced discomfort	Routine infiltration and blocks	[32]
CAD	Controlled flow systems	Precise dosing, reduced tissue trauma	Paediatric and high-sensitivity cases	[35]
USG	Real-time imaging	Accurate needle placement	Complex regional blocks	[36]
Novel Agents	Modified molecular anaesthetics	Lower toxicity, stable clearance	High-risk populations	[30]

Global standards, ethics, and future directions in dental anaesthesia

International guidelines for dental anaesthesia establish shared standards for practitioner training, procedural safety, and patient protection across diverse clinical environments [37]. Regulatory bodies advocate structured frameworks that emphasise competency-based education, standardised dosing principles, comprehensive emergency preparedness, and ongoing professional development [25]. Accreditation systems further specify requirements for facility readiness, including access to appropriate monitoring equipment, airway management devices, and essential pharmacological agents for emergency intervention. By promoting uniform safety benchmarks, these frameworks aim to reduce variability in anaesthetic practice despite differences in healthcare infrastructure and resource availability [28].

Ethical principles underpin anaesthetic decision-making and are guided by patient autonomy, beneficence, non-maleficence, and justice [17]. Informed consent requires clear communication regarding anaesthetic approach, potential risks, procedural steps, and anticipated recovery [29]. Transparent dialogue supports shared decision-making, particularly in high-risk cases or complex interventions. Inequities in access to safe anaesthesia - linked to socioeconomic status, geographic disparities, disability, and institutional limitations - remain an ongoing ethical concern [22].

Future developments in dental anaesthesia are shaped by technological progress, individualised care models, and evolving patient expectations. Integration of real-time physiological monitoring, advanced imaging, and automated drug delivery systems is expected to further improve precision and safety in anaesthetic management [13]. Personalised planning based on genetic, metabolic, and pharmacodynamic profiling offers potential for optimised dosing and reduced adverse events. Expansion of telemonitoring and digital assessment tools may enhance pre-anaesthetic evaluation and postoperative follow-up, particularly in underserved populations [38]. Continued advancement in analgesic agents, airway management technologies, and simulation-based training is likely to strengthen practitioner competence and reduce preventable complications. Alignment with international standards, ethical practice, and responsible adoption of emerging technologies will remain central to improving safety and outcomes in dental anaesthesia across varied clinical settings.

Limitations and future recommendations

This narrative review synthesises evidence derived from studies with diverse methodological designs, resulting in variability in study quality and outcome reporting. The absence of standardised comparative data limits the ability to draw definitive conclusions regarding optimal anaesthetic approaches for specific patient groups. Regional differences in clinical guidelines, monitoring resources, and practitioner expertise further affect the generalisability of findings. Evidence concerning high-risk populations, emerging technologies, and long-term outcomes remains fragmented, restricting the depth of analysis in these areas.

Future research should prioritise high-quality comparative studies evaluating anaesthetic techniques using standardised clinical endpoints, including physiological stability, recovery outcomes, and patient-reported measures. Focused investigation in paediatric, geriatric, and medically complex populations is necessary to strengthen evidence-based anaesthetic decision pathways. Integration of genomic, metabolic, and pharmacodynamic profiling may support the development of more individualised, precision-oriented practice models. Rigorous evaluation of technological innovations, particularly real-time monitoring and automated delivery systems, is required to clarify their impact on safety, efficiency, and clinical applicability across varied dental care settings.

## Conclusions

Local and general anaesthesia remain fundamental components of contemporary dental care and underpin safe, patient-centred practice across a wide range of clinical procedures. Advances in anaesthetic pharmacology, monitoring technologies, and delivery techniques have improved control of anaesthetic depth, reduced complication rates, and enhanced patient experience. Clinical decision-making relies on structured assessment of systemic health, behavioural factors, and procedural requirements to determine the most appropriate anaesthetic modality for each patient. Innovations such as computer-assisted delivery systems, sustained-release formulations, and enhanced physiological monitoring have increased precision and consistency in complex dental interventions. Despite these advances, challenges persist in managing variability in patient responses, addressing the needs of high-risk populations, and ensuring consistent standards across global practice settings. Ethical principles - including informed consent, equitable access to care, and rigorous safety management - continue to guide high-quality anaesthetic practice. Ongoing refinement of techniques, integration of emerging technologies, and systematic evaluation of clinical outcomes are essential to further improve procedural reliability, patient experience, and long-term success in dental anaesthesia.
